# PROJECTING WORKING LIFE EXPECTANCY TO 2050 AMONG OLDER ADULTS IN JAPAN

**DOI:** 10.1093/geroni/igae098.1417

**Published:** 2024-12-31

**Authors:** Abhijit Visaria, Yasuhiko Saito

**Affiliations:** Duke-NUS Medical School, Singapore, Singapore; Nihon University, Tokyo, Japan

## Abstract

With sustained low fertility rate and continued population ageing, the prospect of a shrinking labor force is a key challenge facing Japan. It is of interest to compare two possible scenarios which may result in an increase in the expected years of life spent working (working life expectancy, “WLE”) at older ages: a natural impact of further reductions in mortality, or the impact of extending the mandatory retirement age from 65 to 70 years, as currently being considered by the government. We first estimated WLE among older adults aged 65 years and older using data from five waves of a representative longitudinal survey (1999-2009), applying multistate life table methods. Then, we projected WLE to 2050 by assuming a constant transition schedule between working states (working/not working) but improving age-specific mortality rates (as projected by the UN Population Division). We then simulated the impact of an increase in the mandatory retirement age to 70 years. WLE at age 65 years, based on the longitudinal data, was 4.6 years for men and 3.5 years for women. Improvements in mortality yielded an estimated WLE at the age of 65 in year 2050 of 4.9 years for men and 3.7 years for women. Increasing the retirement age to 70 years increased WLE at age 65 by about 1.5 years for men and 1.6 years for women, by 2050. Our results indicate the value of increasing mandatory retirement age to 70 years as a policy intervention to increase WLE among older adults in Japan.

